# Vascular Endothelial Growth Factor May Be Involved in the Behavioral Changes of Progeny Rats after Exposure to Ceftriaxone Sodium during Pregnancy

**DOI:** 10.4014/jmb.2111.11048

**Published:** 2022-04-22

**Authors:** Xin Yang, Ting Tang, Mengchun Li, Jie Chen, Tingyu Li, Ying Dai, Qian Cheng

**Affiliations:** 1 Department of Primary Child Health Care, Children’s Hospital of Chongqing Medical University, Chongqing 400014, P.R. China; 2 Department of Pediatrics, Daping Hospital, Army Medical University, Chongqing, P.R. China

**Keywords:** Ceftriaxone sodium, *S24-7*, vascular endothelial growth factor

## Abstract

Antibiotic exposure during pregnancy have an adversely effects on offspring behavior and development. However, its mechanism is still poorly understood. To uncover this, we added ceftriaxone sodium to the drinking water of rats during pregnancy and conducted three-chamber sociability test, open-field test, and Morris water maze test in 3- and 6-week-old offspring. The antibiotic group offspring showed lower sociability and spatial learning and memory than control. To determine the role of the gut microbiota and their metabolites in the changes in offspring behavior, fecal samples of 6-week-old offspring rats were sequenced. The composition of dominant gut microbial taxa differed between the control and antibiotic groups. KEGG pathway analysis showed that *S24-7* exerted its effects through the metabolic pathways including mineral absorption, protein digestion and absorption, Valine, leucine, and isoleucine biosynthesis. Correlation analysis showed that *S24-7* abundance was negatively correlated with the level of VEGF, and metabolites associated with *S24-7*-including 3-aminobutanoic acid, dacarbazine, L-leucine, 3-ketosphinganine, 1-methylnicotinamide, and N-acetyl-L-glutamate-were also significantly correlated with VEGF levels. The findings suggest that antibiotic exposure during pregnancy, specifically ceftriaxone sodium, will adversely affects the behavior of offspring rats due to the imbalance of gut microbiota, especially *S24-7*, via VEGF and various metabolic pathways.

## Introduction

Antibiotics have been used in recent years to treat many pregnancy-related diseases [1] and the risk is also increasing [2]. Newborns of mothers who were treated with and without antibiotics during pregnancy show markedly different growth profiles and there is increasing but controversial evidence that exposure to antibiotics during pregnancy may have short-term and long-term effects on babies. The potential behavioral changes of future generations constitute one of the controversies surrounding this phenomenon.

Studies [3-5] suggest that maternal exposure to antibiotics modifies the intestinal microbiome, thus causing maternal immune activation, which may lead to abnormal neurodevelopment and behavioral abnormalities in the offspring. Maternal intestinal microecological disorders may disrupt gut–brain axis of fetus in the uterus and play a role in the occurrence of neurodevelopmental disorders [6, 7]. However, a population cohort study by Hamad *et al*. [8] found no direct association between prenatal antibiotic exposure and increased risk of childhood ADHD. Similarly, Atladóttir *et al*. [9] also did not find that antibiotic use during pregnancy is an important risk factor for autism spectrum disorder (ASD) or infant autism, a disease known to be associated with maternal immune activation. For these reason that effects of antibiotic exposure during pregnancy on offspring neurodevelopment and behavior needs to be further explored.

Antibiotics act through the gut microbiome, maintaining normal physiology in the body through their metabolites [10]. It was also confirmed [11] that antibiotics can alter the structure of the gut microbiome and affect the abundance of metabolites, resulting in a therapeutic effect on necrotizing enterocolitis. Yu *et al*. [12] had found that antibiotic-induced gut microbiome disorders in rats markedly disrupted metabolic pathways related to glycine, serine, and threonine metabolism, niacin and nicotinamide metabolism, and bile acid metabolism. Kimura *et al*. [13] found that the relative abundance of five metabolites differed between specific-pathogen-free (SPF) mice and germ-free (GF) mice during pregnancy, and the plasma levels of short-chain fatty acids (such as acetate, propionate, and butyrate) of offspring were significantly lower in the GF group than in the SPF group.

Based on the above evidence, exposure to antibiotics during pregnancy may have important effects on offspring, especially behavioral changes, which may be caused by an imbalance in the gut microbiome and associated metabolic pathways. But the mechanism remains unclear.

## Materials and Methods

### Experimental Animals

The Sprague-Dawley rats, including SPF-grade female and male, were from the Experimental Animal Center of Chongqing Medical University and kept at the Animal Center of Children's Hospital of Chongqing Medical University (under experimental license SCXK [Yu] 2012-0015). All animal experimental procedures were performed in accordance with the ethical standards set by the ethics committee of the Animal Center of Children's Hospital of Chongqing Medical University.

The male and female rats (1:1 ratio) were housed in a cage overnight, female rats with vaginal plugs the following morning were considered pregnant and recorded as gestational day 01( GD01) , then housed separately. After randomization, pregnant rats were divided into two groups: one of them received ceftriaxone sodium (concentration 1 mg/ml, daily intake is the normal adult dose) [14-16] in their drinking water called antibiotic–pregnant group (*n* = 6) , while the control pregnant group (*n* = 6) did not receive any treatment from GD05 to GD11 of pregnancy. Finally, 50 offspring rats were obtained in _antibiotic group_ and 55 offspring rats were obtained in the control. When study concluded, all of the rats were placed in a closed container and euthanized by using CO_2_.

### Three-Chamber Sociability Test

To find out whether offspring behavior changes, we conducted a three-chamber sociability test to assess the social interaction ability of 3- and 6-week-old offspring rats.

We used three boxes of transparent plastic and placed the rats in the middle box for 5 min one day in advance, allowing them to freely enter and exit. On the following day, formal testing was conducted, a rat of the same age was placed in the left box, a toy was placed in the right box, and the test rats were in the middle box again for 5 min. ANY-maze software and the accompanying camera system automatically recorded the rats’ movement trajectory and time spent in the three boxes.

### Open-Field Test

To assess the independent behavior, exploration ability and tension in the new environment of the offspring, we conducted an open-field test by using a square black plastic box (50 cm × 50 cm × 45 cm), the detection area was artificially divided into nine equal square zones (1 central zone and 8 surrounding zones), and the test rats were placed in the central zone. After 5 min of testing, ANY-maze software and the accompanying camera system automatically recorded the movement trajectory, number of line crossings, and time of activity in the central zone. The video recordings were used to calculate the time spent by each rat on self-grooming.

### Morris Water Maze Test

The Morris water maze was used to the spatial learning and memory ability. A black, circular basin (diameter 130 cm, depth 45 cm) was filled with water until the water level reached 1 cm above a platform. A shade cloth was used to surround the water tank. The shade cloth and the wall of the basin were marked with different shapes to facilitate the identification and memory of direction of the experimental rats. After the experimental rats were gently put in the water near the basin wall, the experimenter quickly left the basin and pulled the shade cloth. ANY-maze software automatically recorded the time taken by the experimental rats to reach the platform and movement trajectory in the water. The entire experimental process lasted 7 days and was divided into three phases of testing and training. On day 1 (adaptation), the platform was removed, and the experimental rats were placed in the water from the first quadrant and removed 1 min later. On day 2–6, positioning navigation training: the platform was fixed in the middle of the third quadrant, 1 cm below the water surface. Ink was added to the water until the naked eye could not see the underwater platform, and the experimental rats from four different initial points-N (north), E (east), S (south), and W (west)-were placed in the water, and the software recorded the trajectory of the experimental rats in the maze. If the platform was not found within 1 min, the experimental rats were guided to the platform for memory enhancement for 10 s, and the incubation period was recorded as 60 s. On day 7 (platform exploration test), the platform was removed again, and the total number of times the experimental rats swam through the original location of the platform in 1 min was recorded.

### Gut Microbiota Sequencing

To determine the effect of gut microbiota, we collected fecal samples for gut microbiota sequencing. Fecal samples were collected from pregnant rats at GD05 and GD12 and from 2-, 3-, and 6-week-old offspring, and the sequencing was done by Shanghai Personal Biotechnology (China). The experiment was done following manufacturer's instructions of the kits used.

After extraction of DNA, the amplification of Target fragment PCR was performed: According to the conserved regions in the target sequence, the corresponding primers were designed incorporating sample-specific barcode sequences, and then the rRNA gene variable region or a specific gene fragment was subjected to PCR amplification.

After the amplification and purification of the amplified product, AXYGEN kit were used to gel recovery the target fragment. PCR amplification products were quantified using the OXYGEN Quant-iT PicoGreen dsDNA Assay Kit and a microplate reader.

Sequencing libraries were prepared by using Illumina's TruSeq Nano DNA LT Library Prep Kit and high-throughput sequencing was performed using a MiSeq sequencer.

### Metabolomics Analysis

To uncover the differences in metabolites, fecal samples from 6-week-old offspring in the two groups were subjected to non-targeted metabolomics analysis, which was based on the ultra-high-performance liquid chromatography (UHPLC) (Shanghai Personal Biotechnology).

Liquid chromatography–tandem mass spectrometry (LC-MS/MS) analysis was used to identify and quantify the metabolites. The samples were separated using an Agilent 1290 Infinity LC Ultra-High-Pressure Liquid Chromatograph (Agilent, Waters Acquity UPLC BEH amide column, 1.7 μm, 2.1 mm × 100 mm). The quadrupole time-of-flight (QTOF) mass spectrometry conditions were as follows: electrospray ionization (ESI) positive and negative ion modes for detection.

The data were uploaded and stored in the SRA(Sequence Read Archive) of NCBI(national center for biotechnology information) repository [SRP319184].

### Detection of Serum Cytokines by Enzyme-Linked Immunosorbent Assay

To detect changes in serum cytokines, serum samples collected at various time points were tested by Shanghai Huaying Biomedical Technology Co., Ltd. The general steps according to the manufacturer’s instructions are as follows:

Sample preparation: The supernatant obtained after centrifugation of the serum was diluted two-fold with the assay buffer. The diluted sample was used for testing.

After standard preparation, serum matrix preparation, chip cytokine/chemokine magnetic bead panel detection operation, sample incubation, Incubate the detection antibody, Read the value into the calibrated Bio-Plex machine and to analysis.

### Statistical Analysis

All statistical operations were performed using GraphPad Prism 7.0 statistical software (GraphPad Software, Inc., USA). The measurement data were expressed as mean ± standard error (x ± SEM). The *t*-test was used to compare the mean values between the two groups and the chi-square test was used to compare the rates. *p* < 0.05 indicated a statistically significant difference.

## Results

### Effect of Antibiotic(ceftriaxone sodium) Exposure during Pregnancy on the Social Behavior and Memory in Offspring

To uncover the effects of antibiotic exposure during pregnancy on behavior and development of offspring, a three-chamber sociability test, open-field test, and Morris water maze test were conducted using offspring rats at 3 and 6 weeks of age. The three-chamber sociability test is used to assess the social communication ability, the open-field test is used to assess the autonomous and exploratory behavior, and the Morris water maze test is used to measure the learning and memory regarding spatial location and positioning.

**Fig. 1:**
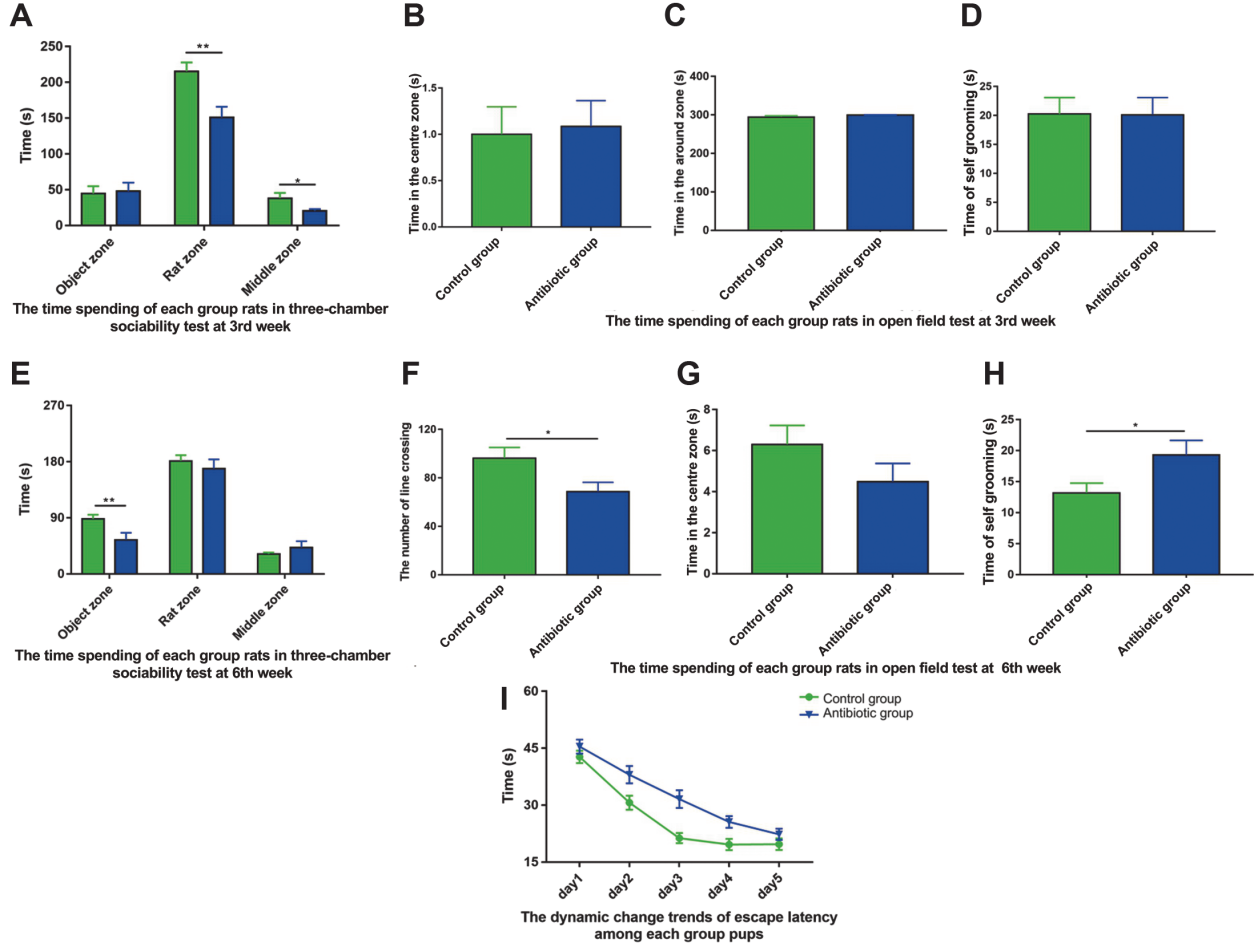
Behavioral experiments conducted with 3- and 6-week-old offspring rats. (**A**) Time spent by 3-week-old rats in the three-chamber sociability test. (**B**) Time spent in the central zone by 3-week-old rats in the open-field test. (**C**) Time spent in the surrounding zones by 3-week-old rats in each group in the open-field test. (**D**) Time spent in self-grooming by 3- week-old rats in each group in the open-field test. (**E**) Time spent by 6-week-old rats in each group in the three-chamber sociability test. (**F**) Number of line crossings by 6-week-old rats in each group in the open-field test. (**G**) Time spent in the central zone by 6-week-old rats in each group in the open-field test. (**H**) Time spent in self-grooming by 6-week-old rats in each group in the open-field test. (**I**) Spatial learning–memory function of offspring rats in each group by the Morris water maze test (n _control group 3rd week_ = 25, n _control group 6th week_ = 30, n _antibiotic group 3rd week_ = 24, n _antibiotic group 6th week_ = 26). Bars represent the mean ± SEM. *p < 0.05, **p < 0.01, ***p < 0.001.

In the three-chamber sociability test, at 3 weeks old, the antibiotic group offspring rats spent significantly less time in the rat zone than the control group offspring rats (*p* < 0.01, Fig. 1A), similarly, at 6 weeks old, the antibiotic group offspring spent significantly less time in the object zone than the control group offspring (*p* < 0.01, Fig. 1E). The time spending In the open-field test, 3-week-old antibiotic and control group offspring rats showed no significant difference in the time spent in the central or surrounding zones or on self-grooming (*p* > 0.05, Figs. 1B–1D). However, at 6 weeks, the number of line crossings of the antibiotic group was significantly lower than that of control (*p* < 0.05, Fig. 1F), and the self-grooming time was significantly higher than that in control (*p* < 0.05, Fig. 1H). In the Morris water maze test, during the positioning navigation training, at week 6, the escape latency in both groups showed a decreasing trend to varying degrees. There was a significant difference between the two groups on days 2, 3, and 4 but no statistical difference in incubation time on day 5. There was no interaction between groups and time points (*p* = 0.108, Fig. 1I). These data indicate that antibiotic exposure during pregnancy decreases the offspring’s social interaction ability, the ability to explore new things, and the spatial learning and memory function.

### Differences in Gut Microbiota Composition between the Two Groups

The gut microbiota was studied by bacterial DNA sequencing and the differences in the gut microbiota composition was investigated using multivariate statistical analysis on the basis of the relative abundance of each bacterial taxon to obtain a variable importance of projection (VIP) score. Statistically significant differences in bacterial taxa abundances were determined using VIP score >1 and independent samples *t*-test (*p* < 0.05).

**Fig. 2:**
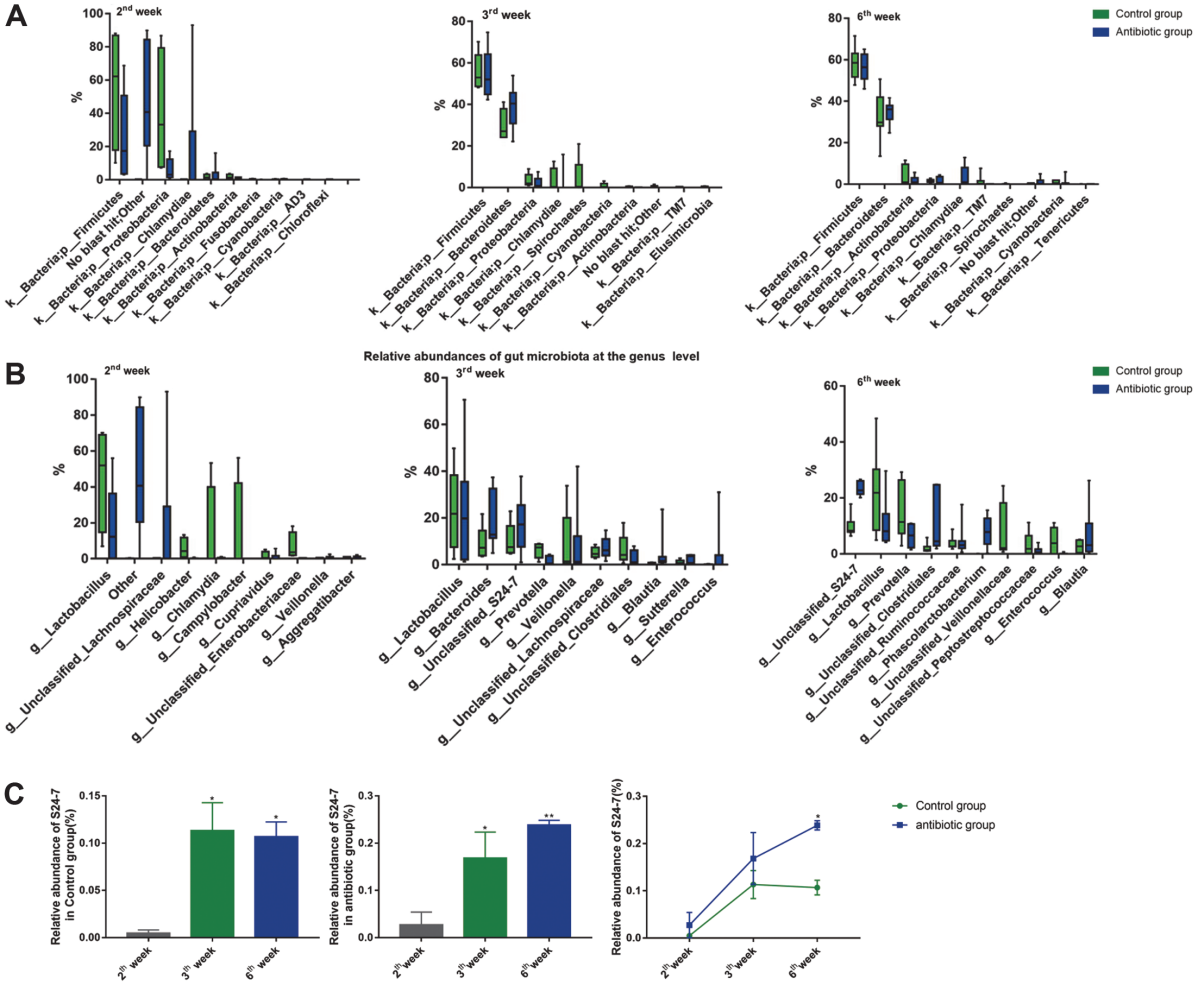
Gut microbiota in the fecal samples of offspring rats. (**A**) Gut microbiota composition at the phylum level. (**B**) Gut microbiota composition at the genus level. (**C**) The relative abundance of *S24-7* in the two groups (n _control group 2nd week_ = 4, n _control group 3rd week_ = 6, n _control group 6th week_ = 7, n _antibiotic group 2nd week_ = 6, n _antibiotic group 3rd week_ = 6, n _antibiotic group 6th week_ = 7). *p < 0.05, **p < 0.01, ***p < 0.001.

In the result of taxonomic composition analysis, in 3-week-old offspring rats, the dominant phyla were Firmicutes (54.8%), Bacteroidetes (39%), Proteobacteria (3.5%), and Chlamydiae (2.6%) in the antibiotic group and Firmicutes (56.9%), Bacteroidetes (30.7%), Spirochaetes (3.7%), and Proteobacteria (3.6%) in the control group. In 6-week-old offspring rats, the dominant phyla were Firmicutes (56.1%), Bacteroidetes (34.7%), and Chlamydiae (3.3%) in the antibiotic group and Firmicutes (58.7%), Bacteroidetes (31.8%), and Actinobacteria (4.7%) in the control group. At 3 weeks, the composition and proportion of predominant bacterial groups was different between the groups. However, at 6 weeks, there was no statistical difference (Fig. 2A).

In 3-week-old offspring rats, the dominant genera in the antibiotic group were *Lachnospiraceae* (7%), *S24-7* (16.8%), *Bacteroides* (20%), and *Lactobacillus* (25.9%). While *Bacteroides* (9.3%), *S24-7* (11.4%), *Veillonella* (14.3%) and *Lactobacillus* (19%) were in control. In 6-week-old offspring rats, the dominant genera were *Clostridiales* (11.2%), *Lactobacillus* (11.5%) and *S24-7* (23.3%) in the antibiotic group and *S24-7* (10%), *Prevotella* (14%) and *Lactobacillus* (23.3%) in control(Fig. 2B). The composition and proportion of predominant bacteria at the genus level were significantly different in both 3- and 6-week-old offspring rats. The relative abundance of *Veillonella* (*p* < 0.05), *Clostridiales* (*p* < 0.05), and *S24-7* (*p* < 0.001) were statistically significantly different between the two groups (Fig. 2B).

To study the differences in gut microbiota, we explored the relative abundance of the significantly different bacterial taxa and found that the relative abundance of *S24-7* at weeks 3 and 6 was significantly higher than that at week 2 in both groups (*p* < 0.05, Fig. 2C), and the relative abundance of *S24-7* in the antibiotic group was higher than that in the control at all time points and significantly higher at week 6 (*p* < 0.05, Fig. 2C). However, the relative abundance of *Clostridiales* and *Veillonella*ceae did not show a significant difference at weeks 3 and 6.

### Differential Metabolites between the Groups

To find out whether the metabolites were different, we used the fecal samples of 6-week-old offspring rats and performed a non-targeted metabolomics analysis and partial least squares discriminant analysis (PLS-DA) was used for multidimensional statistical analysis.

**Fig. 3:**
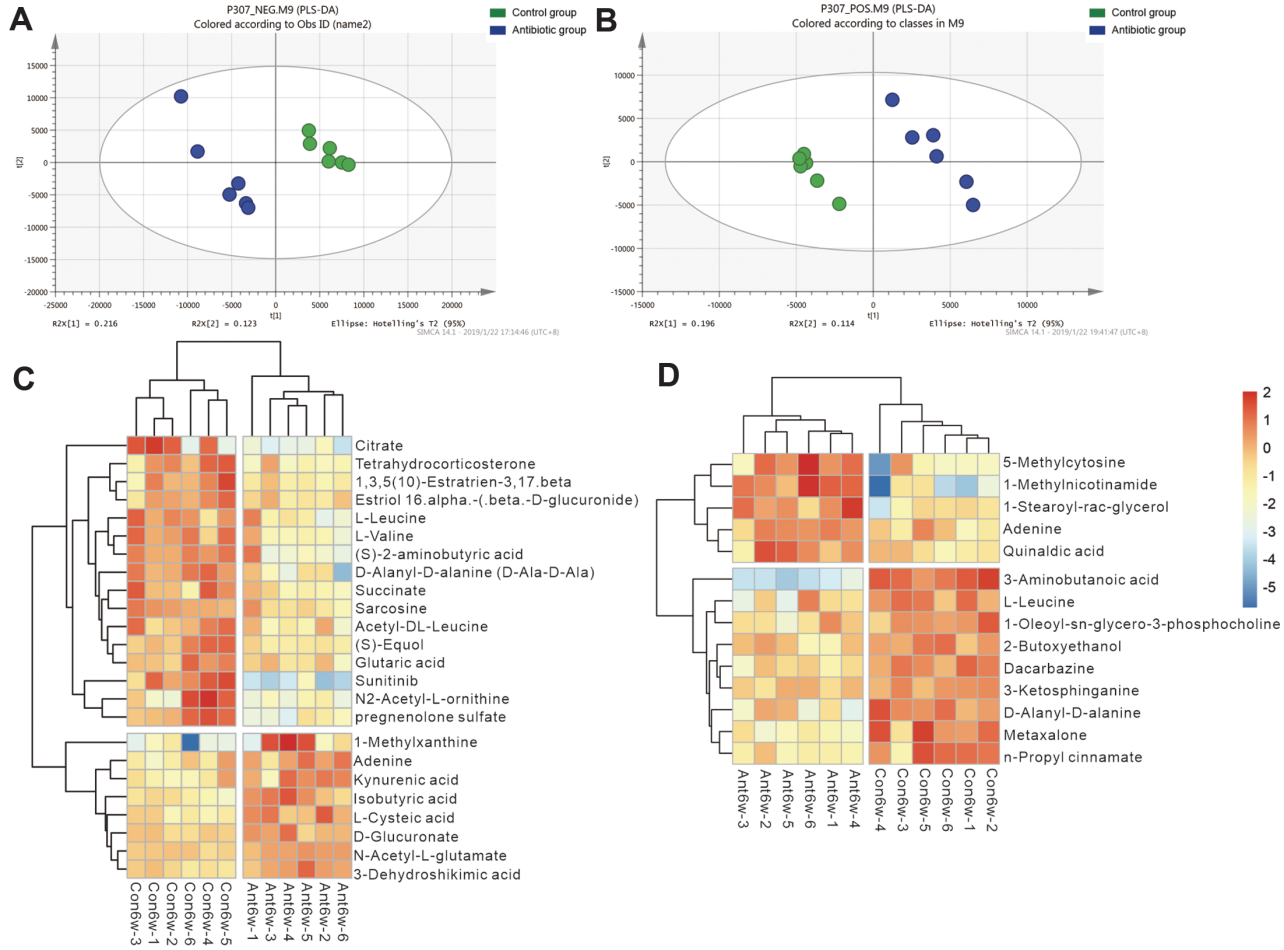
Gut microbiota metabolites extracted from fecal samples of 6-week-old offspring rats were analyzed. (**A**) PLS-DA performed for the anionic metabolites. (**B**) PLS-DA performed for the cationic metabolites. (**C**) Volcano maps constructed for the anionic metabolites in the two groups. (**D**) Volcano maps constructed for the cationic metabolites in the two group (n _control group_ = 6, n _antibiotic group_ = 6).

Used qualitatively significant differences determined using hierarchical clustering of each group of samples to investigate the relationship among samples and the levels of metabolites and accurately screened for marker metabolites. PLS-DA can maximize the difference between groups and reflects t1 and also directly differentiate between groupings. There were differences in the levels of metabolites between the groups as shown in Figs. 3A and 3B. The model orthogonal partial least squares discriminant analysis (OPLS-DA) generates the VIP value. Therefore, we used VIP >1 and an independent sample *t* test (*p* < 0.05) to identify the differentially abundant metabolites.

Among the anionic metabolites, the following metabolites showed lower abundance in the antibiotic group than in the control group in 6-week-old offspring (Ant6w/Con6w, Fig. 3C): sunitinib, L-leucine, estriol 16.alpha.-(.beta.-D-glucuronide) sarcosine, L-valine, D-alanyl-D-alanine, citrate, (S)-equol, pregnenolone sulfate, N2-acetyl-L-ornithine, glutaric acid, succinate, 1,3,5(10)-estratrien-3,17.beta.-diol 17-glucosiduronate, acetyl-DL-leucine, tetrahydrocorticosterone and (S)-2-aminobutyric acid (VIP > 1). The following anionic metabolites showed a higher abundance in the antibiotic group than its in control: L-cysteic acid, kynurenic acid, N-acetyl-L-glutamate, D-glucuronate, isobutyric acid, adenine, 3-dehydroshikimic acid and 1-methylxanthine (VIP > 1).

In the cationic metabolites, the lower abundance in the antibiotic group in 6-week-old offspring (Ant6w/Con6w, Fig. 3D) were as follows: metaxalone, D-alanyl-D-alanine, 3-ketosphinganine, n-propyl cinnamate, 2-butoxyethanol, L-leucine, 3-aminobutanoic acid, 1-oleoyl-sn-glycero-3-phosphocholine, and dacarbazine (VIP > 1). The higher abundance in the antibiotic group were as follows: 5-methylcytosine, 1-stearoyl-rac-glycerol, adenine, 1-methylnicotinamide, and quinaldic acid (VIP > 1).

### Relationship between Differential Metabolites and Gut Microbiota

**Fig. 4:**
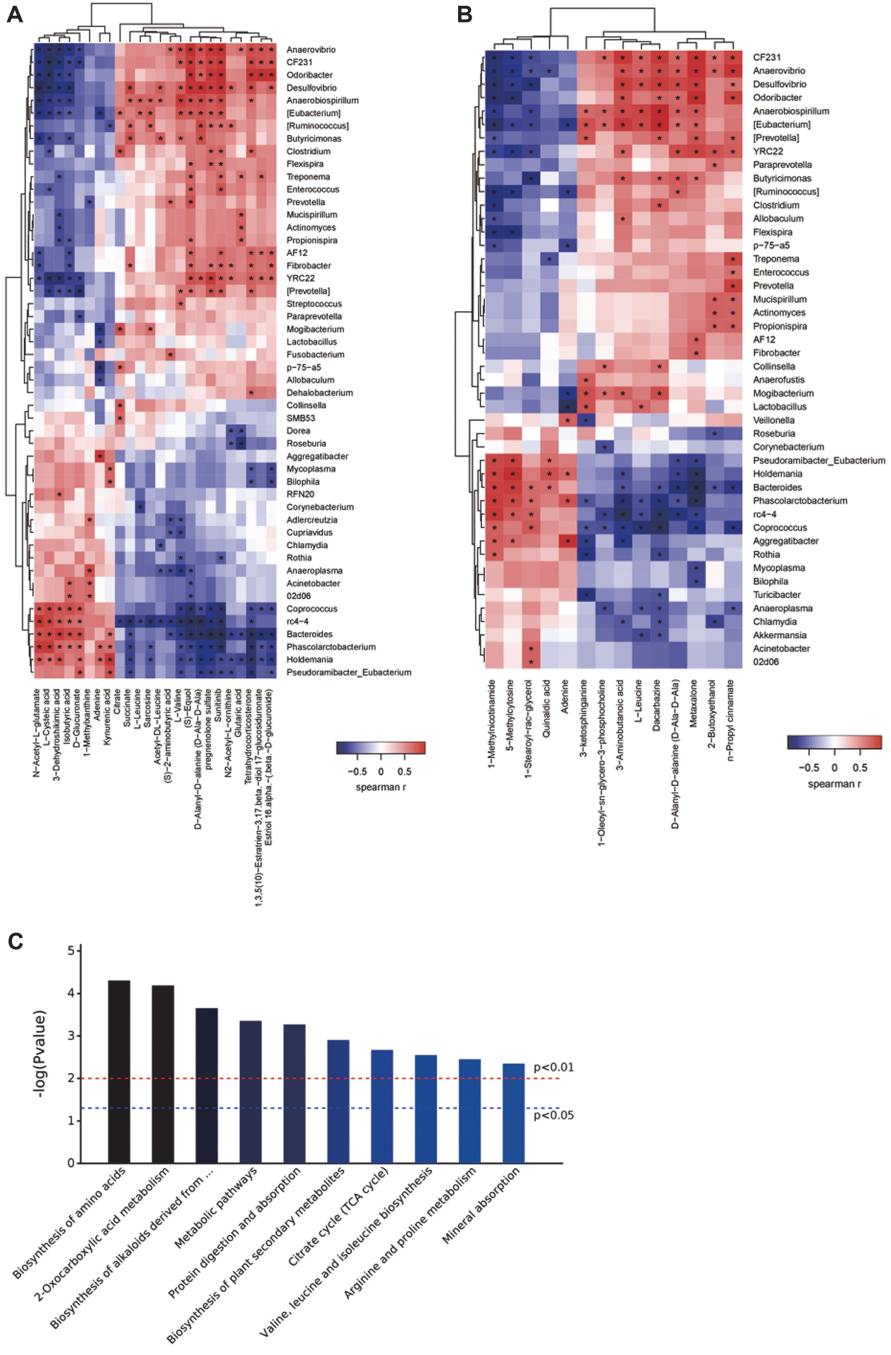
Relationship between metabolites and gut microbiota. (**A**) Heat map of the correlation between the gut microbial species and anionic metabolites. Positive and negative correlations are depicted in red and blue, respectively. The shade of the color indicates the strength of the correlation. * indicates the significantly correlated species–metabolite pairs (*p* < 0.05). (**B**) Heat map of the correlation between the gut microbial species and cationic metabolites. (**C**) Differential metabolites identified (including positive and negative ion mode results) were subjected to the Kyoto Encyclopedia of Genes and Genomes (KEGG) pathway analysis (n _control group_ = 6, n _antibiotic group_ = 6).

The relationship between differential metabolites and gut microbiota was studied using Mothur software to calculate the Spearman rank correlation coefficient. According to the correlation coefficient matrix results (rho correlation coefficient is a value between -1 and 1, -1 < rho < 0 indicates a negative correlation, 0 < rho < 1 indicates a positive correlation, rho = 0 indicates no correlation), a heat map was drawn using R software (Figs. 4A and 4B).

We subjected the identified differentially abundant metabolites to a KEGG pathway analysis (Fig. 4C) and the results showed that the metabolic pathways in the feces including: Protein digestion and absorption, Metabolic pathways, Mineral absorption,Valine, leucine and isoleucine biosynthesis, and Citrate cycle (TCA cycle) etc.in the two groups changed significantly.

### Differences in Cytokine Levels between Groups

**Fig. 5:**
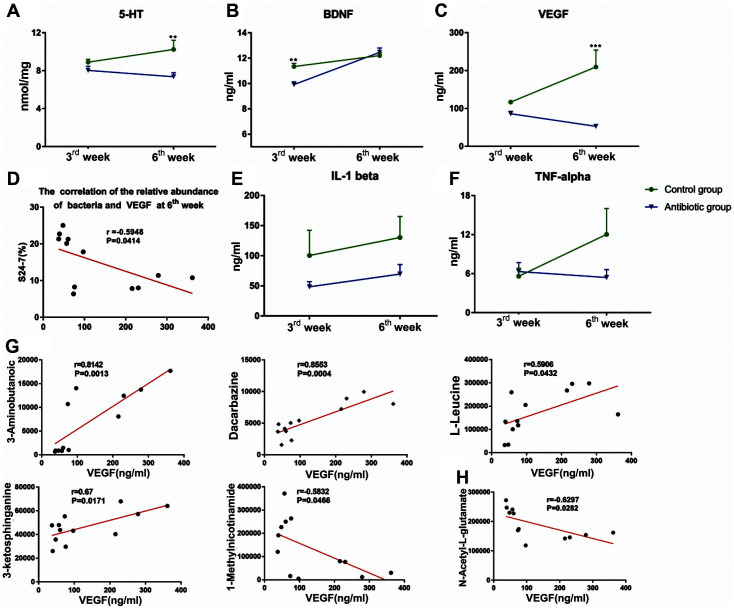
Detection of the serum levels of cytokines in 3- and 6-week-old offspring rats in the antibiotic and control groups. (**A**) Serum levels of 5-TH in the two groups. (**B**) Serum levels of BDNF in the two groups. (**C**) Serum levels of VEGF in the two groups. (**D**) Relationship between the relative abundance of *S24-7* and VEGF serum levels. (**E**) Serum levels of IL-1β in the two groups. (**F**) Serum levels of TNF-α in the two groups. (**G**) Relationship between VEGF levels and cationic metabolites. (**D**) Relationship between VEGF levels and anionic metabolites (n _Control group 3rd week_ = 6, n _Control group 6th week_ = 9, n _antibiotic group 3rd week_ = 6, n _antibiotic group 6th week_ = 7). **p < 0.01, ***P < 0.001.

To study the mechanism underlying the behavioral changes of the offspring rats, we performed ELISA using serum samples collected from 3- and 6-week-old offspring rats. In 3-week-old offspring rats, no significant differences were observed in the cytokine levels of the two groups except brain-derived neurotrophic factor (BDNF), which showed significantly higher levels in the antibiotic group than in control (*p* < 0.01, Fig. 5B). In 6-week-old offspring rats, BDNF levels were not significantly different between the two groups. However, the levels of 5-TH (*p* < 0.01, Fig. 5A) and vascular endothelial growth factor (VEGF, *p* < 0.001, Fig. 5C) in the antibiotic group were significantly lower than those in control.

To find out the relationship between the special one S24-7 and cytokines, we conducted a correlation analysis and the result showed that the abundance of *S24-7* showed a significant negative correlation with the levels of VEGF (*p* < 0.05, Fig. 5D). Moreover, we reanalyzed the relationship between VEGF and certain metabolites associated with *S24-7* and found that some metabolites-including 3-aminobutanoic, dacarbazine, L-leucine, 3-ketosphinganine, 1-methylnicotinamide and N-acetyl-L-glutamate—are also significantly correlated with VEGF levels (Figs. 5G and 5H).

## Discussion

### Antibiotic Exposure in Pregnancy Affects Offspring Behavior

Studies have shown that approximately 25% women during pregnancy are prescribed antibiotics, accounting for approximately 80% of drugs prescribed to pregnant women, especially in the first and second trimesters owing to infection, inflammation, or other high-risk factors [17-19]. The β-lactam antibiotic ceftriaxone sodium, an effective therapeutic drug, has been found to induce distinct side effects. A recent study of adverse drug reactions in pregnant women showed that the incidence of immune-related adverse events associated with ceftriaxone sodium was 18.4% [20], which may result in gut microbiota imbalance because of changes in the abundance of dominant taxa. Previously, we added ceftriaxone sodium to the drinking water of Sprague–Dawley rats during pregnancy and found the effect of ceftriaxone sodium on the weight of offspring, bacterial DNA sequencing also showed that structure of gut microbiota was markedly disrupted in the pregnant rats [21].

In the present study, the pregnant rats to ceftriaxone sodium by adding it to the drinking water and subjected 3-and 6-week-old offspring rats to a open-field test, three-chamber sociability test, and Morris water maze test. We found that the offspring in the antibiotic group showed a lower social interaction ability and spatial learning and memory function than the offspring in the control group. Moreover, these behavioral changes become more pronounced with increasing age. Degroote *et al*. [22] found that the offspring who were exposed to antibiotics during the perinatal period spent approximately 50% less time on social interaction than the control group offspring. They concluded that antibiotic exposure seriously interferes with the intestinal flora of the offspring, which is consistent with our findings. Similarly, Vuong *et al*. [23] had found changes in maternal gut microbiome affect the neurobehavioral response of offspring, which may be achieved by sending signals to developing neurons through microbial-regulated metabolites.

### Different Gut Microbiota Composition Contributes to Behavioral Changes of Offspring

To explore whether antibiotic exposure during pregnancy affected the gut microbiota of the offspring, we performed bacterial DNA sequencing using fecal samples of the offspring.

The alpha-diversity analysis showed no significant difference between the control group and the antibiotic exposure group. However, the beta-diversity analysis showed significant differences in the gut microbiota structure of the two groups. These results have been published before [21, 24].

The results showed that the dominant bacteria at the phylum level in the 3-week-old offspring rats were mainly Firmicutes and Bacteroidetes in the control and antibiotic groups. However, the abundance varied between groups. In 6-week-old offspring rats, the dominant phyla were Firmicutes and Bacteroidetes in control, Firmicutes, Bacteroidetes and Chlamydiae in the antibiotic group. According to a previous study, Firmicutes and Pseudobacteria are the predominant phyla in the gut microbiota of rats [25]. Other studies have found that the abundance of the phyla Bacteroidetes and Firmicutes in offspring rats changed after a high-fat diet, accompanied by behavioral changes [26]. Similarly, it was found that, in children with autism, the relative abundance of Bacteroidetes reduced, and the ratio of Firmicutes/Bacteroidetes significantly increased [27].

The dominant gut bacteria at the genus level in the two groups were similar but also with differences, the relative abundance of *S24-7* in the antibiotic group offspring rats gradually increased and was significantly different from that in control. Benakis *et al*. [28] found that *S24-7* is involved in neurological diseases such as ischemic stroke in mice. An analysis of the effects of light stress showed that the relative abundance of *S24-7* was negatively correlated with increasing light-dark stress, which was associated with changes in motor and memory behavior [29]. Another study found that gut microbiota transplantation improves the behavioral abnormalities of mouse models of ASD and significantly reduced the relative abundance of key differential taxa, including *S24-7* [30], which also supports our findings. Therefore, the present findings suggest that gut microbiota, particularly *S24-7*, is involved in the behavioral changes of the offspring.

### Different Metabolites May Affect the Behavioral Changes of Offspring

Non-targeted metabolomics analysis identified the differentially abundant metabolites in 6-week-old offspring rats between the two groups: N2-acetyl-L-ornithine, sunitinib, citrate, and etc. Previous studies [31] have found that bacterially produced metabolites such as valine, isoleucine, acetate are involved in amino acid metabolism, energy metabolism, especially endocrine metabolism, lipid metabolism. Moreover, several plasma metabolites, including glycolic acid, tryptophan, and pyruvate are involved in behavioral changes [30], and homovanillic acid, glutamate, lactate are involved in awakening behaviors [32].

### S24-7 May Affect the Behavioral Changes of Offspring through VEGF and Different Metabolic Pathways

The correlation analysis identified several anionic and cationic metabolites correlated with the abundance of certain gut microbiota. The KEGG pathway analysis results suggest that metabolic pathways of offspring was affected by antibiotic exposure during pregnancy. Protein synthesis and glucose homeostasis are affected by branched-chain amino acids, including isoleucine, leucine, and valine [33]. Valine and leucine is also involved in metabolic processes. Oral administration of β-alanyl-L-leucine in rats leads to changes in behavior [34]. Protein digestion and absorption, mineral absorption, and the citrate cycle also affect metabolism, which explains the behavioral changes of the offspring in the present study. These pathways may represent the mechanism of gut microbiota exert biological effects via metabolites.

Previous studies showed that the behavioral changes in offspring are related to the maternal immune activation following antibiotic exposure during pregnancy. However, the present study found no significant difference in the serum levels of IL-1β and TNF-α but found a difference in the serum levels of 5-TH, BDNF, and VEGF between the antibiotic and control group offspring. Moreover, the abundance of *S24-7* was found to be significantly negatively correlated with VEGF levels. Previous studies show that VEGF, a disulfide-bonded glycoprotein dimer promotes endothelial growth, is an important factor in angiogenesis, and has neurotrophic effects on glial and neuronal cells [35]. VEGF treatment also improves the depression-like behavior of adult offspring due to maternal immune activation [36]. These findings suggest that *S24-7* contributed to the behavioral changes of offspring rats by regulating the level of VEGF. In the present study, no correlation was found between *S24-7* and the levels of BDNF or 5-TH. However, previous studies have found that some bacteria (including *Lactobacillus* and *Bifidobacterium*) produce neurotransmitters such as γ-aminobutyric acid and catecholamines [37], which also opened up our thinking for our experiment.

Similarly, *S24-7* has been found to be related to leucine, L-valine, and glutaric acid. L-valine attenuated paraquat-induced motor behavior and neurochemical disorders in vivo [38], whereas leucine prevented lipopolysaccharide-induced depression-like behavior in mice [39]. It also observed [40] that the abundance of *S24-7* was negatively correlated with L-valine and L-leucine levels, which is consistent with the present findings. Dacarbazine, an important anti-angiogenic agent, is often found to be correlated with VEGF [41, 42]. Leucine-rich α-2-glycoprotein, a kind of novel pro-angiogenic factor, has an important synergistic role with VEGF in maintaining renal function [43]. Similarly, the present study found that the levels of these kinds of metabolites were significantly correlated with *S24-7* abundance and also closely associated with VEGF levels.

In summary, our findings suggest that exposure to antibiotics during pregnancy can have a long-lasting adverse effect on offspring behavior. After ceftriaxone sodium exposure, colonization by different gut microbiota, especially *S24-7*, may affect offspring behavior through different metabolic pathways. However, further research is required to understand the detailed mechanism underlying how *S24-7* exerts its effects through metabolites and the relationship between intestinal flora, metabolites, VEGF, and other cytokines.
